# Effects of Inoculants Producing Antifungal and Carboxylesterase Activities on Corn Silage and Its Shelf Life against Mold Contamination at Feed-Out Phase

**DOI:** 10.3390/microorganisms9030558

**Published:** 2021-03-08

**Authors:** Dimas Hand Vidya Paradhipta, Young Ho Joo, Hyuk Jun Lee, Seong Shin Lee, Hyeon Tak Noh, Jeong Seok Choi, Jinwoo Kim, Hyeong Gyu Min, Sam Churl Kim

**Affiliations:** 1 Division of Applied Life Science (BK21Four, Institute of Agriculture & Life Science), Gyeongsang National University, Jinju 52828, Korea; dimazhand@gmail.com (D.H.V.P.); hyukjun0209@gmail.com (H.J.L.); seongshin73@gmail.com (S.S.L.); nht1647@gmail.com (H.T.N.); x47677104@gmail.com (J.S.C.); 2 Faculty of Animal Science, Universitas Gadjah Mada, Yogyakarta 55281, Indonesia; 3 Institute of Agriculture and Life Science, Gyeongsang National University, Jinju 52828, Korea; wn5886@gmail.com; 4 Department of Plant Medicine, Gyeongsang National University, Jinju 52828, Korea; jinwoo@gnu.ac.kr; 5 Agricultural Promotion Department, Sancheong Agricultural Technology Center, Sancheong 52221, Korea; minhg@korea.kr

**Keywords:** antifungal, carboxylesterase, corn silage, feed-out phase, *Fusarium graminearum*, *Lactobacillus brevis* 5M2, *Lactobacillus buchneri* 6M1

## Abstract

The present study aimed to investigate effects of dual-purpose inoculants (antifungal and carboxylesterase activities) not only on corn silage quality, but also its shelf life against mold contamination at feed-out phase. Corn forage was ensiled for 252 d with different inoculants of the following: control (CON), *Lactobacillus brevis* 5M2 (5M), *Lactobacillus buchneri* 6M1 (6M), and mixture of 5M and 6M at 1:1 ratio (MIX). After ensiling, corn silage was contaminated with *Fusarium graminearum*. Silages applied inoculants had positive effects by increased organic acid and lactic acid bacteria, and decreased undesirable microbes. At feed-out phase, contamination of *F. graminearum* into corn silage had a negative effect on aerobic stability caused by increased growth of undesirable microbes. However, silages applied inoculants had positive effects by decreased undesirable microbes and extended lactic acid bacteria and aerobic stability. Generally, MIX silage presented better effects on organic acid production, rumen degradation, inhibition of undesirable microbes, and aerobic stability than 5M silage and 6M silage. The present study concluded that application of inoculants into corn silage had positive effects on fermentation characteristics and extended shelf life against mold contamination at feed-out phase. A mixed inoculant appeared to have better effects of antifungal and carboxylesterase than a single inoculant.

## 1. Introduction

Whole crop corn is the major cereal forage that often used as silage to supply the requirement of dietary fiber for both beef and dairy industries [1]. However, low fermentation quality and digestibility on corn silage can occur due to the contamination of undesirable microbes and lignification. In the last decade, many studies have been conducted to increase the quality of corn silage by applying microbial additives [2,3]. Previous researchers discovered that several species of lactic acid bacteria (LAB) could produce not only lactate, but also antifungal substances, which supported their roles as probiotics to improve animal health [4]. Additionally, the other studies reported that several species of LAB could release fibrolytic enzymes, such as carboxylesterase, which could improve the digestibility in the rumen [5]. Application of these LAB as silage inoculants have been proven to present antifungal effects by improving aerobic stability [3,6,7] and carboxylesterase effect by increasing rumen digestibility [3,8,9]. Recently, the use of LAB as silage additive is strongly recommended by many previous studies [2,3,5,6,7,8,9] because LAB not only improves ensiling quality, but also potentially has a positive interaction with rumen microbes to increase rumen fermentation [5,8]. Moreover, Muck et al. [5] reviewed that the silage inoculated with LAB still presented beneficial effects as probiotic on ruminant performance and health.

In the viewpoint of field practice, contaminations of undesirable microbes such as yeast and mold on corn silage can occur in all steps of making silage consisting of preharvest, storage, and after fermentation/feed-out phases [10,11]. Keller et al. [12] reported that corn forage just before ensiling contained higher mycotoxins concentration than corn silage. It indicated that the contamination of undesirable microbes can be started in the field, such as rust infestation on the corn plant [10,13]. In addition, several factors during storage consisting of high temperature, low density, and broken silo can be the other causes for the increased contamination of undesirable microbes in the silage [11,14]. At feed-out phase, longer time of aerobic exposure decreases shelf life of silage. Commonly, in the small farm scale, baled silage can be exposed to the air for 3–5 d during feed-out. Mold mycotoxins infect silage aerobically and the damp weather can be a factor to accelerate its infection in the farm [15]. The environmental conditions of farm including humidity, temperature, and climate are uncontrolled factors and strongly influence the growth of mycotoxin molds that can affect shelf life of silage [10,11]. Besides environmental condition, contamination of mold on corn silage may occur when corn silage is mixed with other ingredients contaminating mycotoxins due to a poor management of feed storage. Silage study at preharvest and storage phase was conducted in many previous studies [3,10,11]. However, silage study at feed-out phase, especially against mold contamination, is in limited investigation.

In our previous study [16], *Lactobacillus brevis* 5M2 KACC 92268P and *L. buchneri* 6M1 KACC 92269P were screened from corn silage and selected based on their ability to present antifungal activity by inhibiting the growth of *Fusarium graminearum* and fibrolytic activity by producing carboxylesterase. However, the effects of these dual-purpose inoculants on shelf life of corn silage against mold contamination at feed out phase were not investigated yet. To demonstrate the mold contamination at feed-out phase, *F. graminearum* was applied into corn silage just after silo open in the present study. The *F. graminearum* are the most abundant mycotoxin mold of corn plants grown in South Korea [17]. It was hypothesized that antifungal activity of inoculant could extend shelf life of corn silage against mold contamination at feed-out phase, while carboxylesterase activity of inoculant could improve rumen degradation of corn silage. Therefore, the aim of present study determined effects of dual-purpose inoculants producing antifungal substances and carboxylesterase not only on chemical composition, fermentation characteristics, and rumen degradation of corn silage, but also its shelf life against the contamination of mold at feed-out phase.

## 2. Materials and Methods

### 2.1. Inoculant Preparation

Both LAB, consisting of *L. brevis* 5M2 KACC 92268P and *L. buchneri* 6M1 KACC 92269P, were grown in lactobacilli de Man Rogosa Sharpe media (MRS; Difco, Detroit, MI, USA) for 48 h in a CO_2_ incubator (Thermo Scientific, Waltham, MA, USA), and then diluted with sterile ultra-pure distilled water to reach the recommended application rate at 1 × 105 colony forming unit (cfu)/g according to several previous studies [3,18,19].

### 2.2. Silage Production

Whole crop corn (*Kwangpyeongok* hybrid) was sown at 10 ha of a cornfield at Sancheong Agricultural Research and Extension Service, Sancheong, Gyeongnam province, South Korea (latitude 35°18′ N and longitude 127°56′ E). Corn forage was harvested at ½ milk line stage, and then chopped into 3–5 cm length using a Claas of Jaguar 850 corn harvester (Claas of America, Columbus, IN, USA). The chopped corn forage was ensiled into a 20 L mini silo (8 kg) for 252 d with different inoculants of the following: (i) no inoculant, applied distilled water at 80 µL/g of fresh forage (CON); (ii) *L. brevis* 5M2 KACC 92268P, applied at 1 × 10^5^ cfu/g of fresh forage (5M); (iii) *L. buchneri* 6M1 KACC 92269P, applied at 1 × 10^5^ cfu/g of fresh forage (6M); (iv) mixture of *L. brevis* 5M2 KACC 92268P and *L. buchneri* 6M1 KACC 92269P at 1:1 ratio, applied at 1 × 10^5^ cfu/g of fresh forage (MIX). Each inoculant treatment, including CON silage, used four silos as replications. The distilled water and all inoculants were sprayed individually for each silo. During ensiling, all silos were stored at room temperature (20 ± 2 °C).

The fresh forage just before ensiling was subsampled from each silo, and then composited for each treatment at 500 g. Furthermore, silages from 16 silos were also subsampled at 500 g, respectively. The composited fresh forage and subsampled silage were used for analyses of chemical compositions and in vitro digestibility. To make silage extraction, 20 g of silage was subsampled from each silo and blended with sterile ultrapure water at 200 mL of during 30 s [16,20]. The mixture of silage and ultrapure water was filtered through two layers of cheesecloth [16,20]. After filtering, the fresh silage extraction was used to analyze pH and microbial counts. For the further analyses of fermentation characteristics, silage extraction was stored at −70 °C until. In addition, silage was subsampled at 2 kg and placed in aerobic condition to measure the aerobic stability.

### 2.3. Chemical Compositions

The subsampled forage and silage were placed into dry oven (OF-22GW, Jeio Tech, Seoul, South Korea) at 65 °C for 48 h. Then, samples were ground using a cutting mill (Shinmyung Electric Co., Ltd., Gimpo, South Korea) to pass 1-mm screen as a recommended size to measure chemical compositions and in vitro rumen digestibility. Samples at 10 g were dried in a forced-draft oven (OF-22GW, Jeio Tech, Seoul, South Korea) at 105 °C for 24 h (method 934.01 of AOAC [21]) to measure dry matter (DM). Crude ash (CA) was determined with a muffle furnace at 550 °C for 5 h (method 942.05 of AOAC [21]). Crude protein (CP) was measured by procedure of Kjeldahl (method 984.13 of AOAC [21]) through N analyzer (B-324, 412, 435 and 719 S Titrino, BUCHI, Flawil, Switzerland). On the other side, ether extract (EE) was determined by the producer of Soxhlet (method 920.39 of AOAC [21]). An Ankom^200^ Fiber Analyzer (Ankom Technology, Macedon, NY, USA) was used to analyze neutral detergent fiber (NDF; method 2002.04) and acid detergent fiber (ADF; method 973.13) according to protocol of AOAC [21]. The NDF was analyzed with heat stable amylase. Both NDF and ADF were expressed inclusive of residual ash. The difference concentration between NDF and ADF was determined as Hemicellulose (HEMI). The in vitro DM digestibility (IVDMD) was determined after incubating a dried sample (0.5 g) with rumen buffer for 48 h described by Tilley and Terry [22] using an Ankom Daisy^II^ (Ankom Technology, Macedon, NY, USA). Residue from the IVDMD assay was analyzed NDF to determine in vitro NDF digestibility (IVNDFD). Then, both the results of IVDMD and the IVNDFD were expressed as a percentage of DM (g/kg DM).

### 2.4. Fermentation Characteristics

Silage pH was measured using a pH meter (SevenEasy, Mettler Toledo, Greifensee, Switzerland), while ammonia-N was determined by the colorimetric method following the protocol of Chaney and Marbach [23]. Before analyzing lactate and volatile fatty acid (VFA), the centrifugation of silage extraction at 5645× *g* for 15 min was conducted to collect the supernatant [16,24]. The supernatant was used to measure the concentrations of lactate and VFA using HPLC (L-2200, Hitachi, Tokyo, Japan) fitted with a UV detector (L-2400, Hitachi, Tokyo, Japan) and a column (Metacarb 87H, Varian, Palo Alto, CA, USA) following the method of Muck and Dickerson [25].

### 2.5. Microbial Counts

Fresh silage extract was continued into 10^−2^–10^−8^ of dilution series to analyze microbial counts such as LAB, yeast, and mold. Different selective medium was prepared for each microbe. The LAB was grown at lactobacilli MRS agar media, while yeast and mold were grown at potato dextrose agar media (PDA; Difco, Detroit, MI, USA). The silage extract with different dilution series was plated in each selective agar medium with three replications, respectively. Furthermore, the CO_2_ incubator (Thermo Scientific, Waltham, MA, USA) was used to store MRS agar at 28 °C for 48 h [16]. On the other side, aerobic incubator (Johnsam Corp., Boocheon, South Korea) was used to store PDA plates at 28 °C for 72 h [16]. Visible colonies from each plate were counted. The number of cfu was presented per gram of silage. The result of microbial data was transformed to log10.

### 2.6. Rumen Degradation Kinetics and Fermentation Indices

The animal care and procedure to maintain a cannulated heifer was approved by animal ethical committee of Gyeongsang National University, Jinju, South Korea (GNU-191011-E0050). Before morning feeding, the rumen fluid (pH 6.68) was collected and composited from two non-pregnant cannulated heifers. The diet of cannulated heifers consisted of rice straw and commercial concentrate mix at 8:2 ratio with addition of vitamin-mineral premix. Furthermore, rumen fluid was filtered with two layers of cheesecloth to reduce remains diet in the fluid. Anaerobic culture medium was prepared according to previous study, and then mixed with rumen fluid at 1:2 ratio to make rumen buffer [26]. Dried sample at 0.5 g was prepared in an incubation bottle with 40 mL of rumen buffer. To reach anaerobic condition, the incubation bottle was gassed with CO_2_ and closed tightly with gas production sensor by ANKOM^RF^ (Ankom Technology, Macedon, NY, USA). A total of 16 samples from each silo were incubated into 16 bottles along with two blanks. All incubation bottles were stored in an incubator at 39 °C for 72 h. Gas pressure was recorded in a computer at every 30 min also following the method of Adesogan et al. [26] to determine rumen degradation kinetics. At 10 psi of gas pressure in the incubation bottle, the sensor automatically opened the vent to release the gas. Calculation of rumen degradation kinetics used PROC NLIN of Statistical Analysis Software (SAS; Version 9. Cary, NC, USA) [27] to fit with the model of McDonald [28] following:

Y = A + B (1 − e^−c(t−L)^) for t > L

where A is the immediately degradable fraction; B is the potentially degradable fraction; A + B is the total degradable fraction; C is the fractional degradation rate; L is lag phase; t is time of incubation (h).

Each incubation bottle was opened after 72 h of incubation. The mixture of sample and rumen buffer from incubation bottle was transferred to 50 mL conical tube. Furthermore, the centrifugation at 2568× *g* for 15 min (Supra 21k, Hanil Electric Corporation, Seoul, South Korea, with rotor A50S-6C No.6) was conducted to separate remains sample and supernatant (rumen buffer). Both remains sample and supernatant were collected for further analyses. The supernatant was used to determine rumen fermentation indices consisting of pH, ammonia-N, and VFA. The protocols to analyze pH, VFA, and ammonia-N were the same as described above.

### 2.7. Microbial Changes and Aerobic Stability at Feed-Out Phase

In aerobic condition, 1 kg of subsampled silage from each silo was placed in a box of polystyrene and stored for 120 h at room temperature (22 ± 2 °C). Two sensors of thermocouple (MORGAN TR-60CH, Hong Kong, China) were placed at the geometric center of each sample to record silage temperature. In the present study, temperature data of silage and room were collected every 30 min. In principle, aerobic stability was measured by the time (h) before a 2 °C increase in silage temperature above the ambient temperature [20]. The other 1 kg of subsampled silage from each silo was applied with *F. graminearum* head blight fungus MHGNU F312 at 1 × 10^5^ cfu/g of fresh forage before measure the aerobic stability. The application rate of *F. graminearum* in the present study was conducted based on actual mold count of corn silages at a farm in our previous investigation [16]. Previously, *F. graminearum* head blight fungus MHGNU F312 was obtained through isolation procedure from the diseased corn plants. Before applied into corn silage, *F. graminearum* were grown in potato dextrose for 72 h in aerobe incubator (Johnsam Corp., Boocheon, South Korea), and then diluted with sterile ultra-pure distilled water to reach the recommended application rate. Generally, silages without *F. graminearum* application were labeled as ‘without contamination’, while silages with *F. graminearum* application were labeled as ‘with contamination’. In this measurement, 32 polystyrene boxes were prepared to allocate silages without and with contaminations. All polystyrene boxes were stored in the same room condition. In addition, 50 g of silage from each polystyrene box were subsampled at 24, 48, 72, 96, and 120 h of aerobic exposure to measure the microbial counts. The protocol for counting LAB, yeast, and mold were the same as described above.

### 2.8. Statistical Analysis

All collected data in the present study were statistically analyzed as a completely randomized design by PROC GLM of SAS [27]. Its general model was *Yij* = *µ* + *Ti* + *eij*, where *Yij* = response variable, *µ* = overall mean, *Ti* = the effect of inoculant, *eij* = error term. For microbial counts at feed-out phase, data were statistically analyzed as a factorial design with a 4 (Inoculant; CON vs. 5M vs. 6M vs. MIX) × 2 (*Fusarium*; without contamination vs. with contamination) × 6 (aerobic hour; 0 vs. 24 vs. 48 vs. 72 vs. 96 vs. 120 h) arrangement by PROC MIX of SAS (SAS 2002). Its general model was *Yijkl* = *µ* + *αi* + *βj* + Ɣ*k* + *(αβ)ij* + *(α*Ɣ*)ik* + *(β*Ɣ*)ik* + *(αβ*Ɣ*)ijk* + *eijkl*, where *Yijkl* = response variable, *µ* = overall mean, *αi* = the effect of inoculant, *βj* = the effect of *Fusarium*, Ɣ*k* = the effect of aerobic hour, *(αβ)ij* = the interaction effect of inoculant and *Fusarium*, *(α*Ɣ*)ik* = the interaction effect of inoculant and aerobic hour, *(β*Ɣ*)jk* = the interaction effect of *Fusarium* and aerobic hour, *(αβ*Ɣ*)ijk* = the interaction effect of inoculant, *Fusarium*, and aerobic hour, *eijkl* = error term. In addition, polynomial contrast through PROC GLM of SAS was used to investigate the linear or quadratic effect of aerobic hour on microbial counts at feed-out phase. Before testing a polynomial contrast for significance, the orthogonal coefficients for linear and quadratic contrasts were adjusted to account the spacing of aerobic hour (0, 24, 48, 72, 96, and 120 h) with PROC IML of SAS [27] For the aerobic stability, data was statistically analyzed as a factorial design with a 4 (Inoculant) × 2 (*Fusarium*) arrangement by PROC MIXED of SAS [27]. Its general model was *Yijk* = *µ* + *αi* + *βj* + *(αβ)ij* + *eijk*, where *Yijk* = response variable, *µ* = overall mean, *αi* = the effect of inoculant, *βj* = the effect of *Fusarium*, *(αβ)ij* = the interaction effect of inoculant and *Fusarium*, *eijk* = error term. Mean separation was conducted by Tukey’s test and the significant differences were declared at *p* ≤ 0.05.

## 3. Results

### 3.1. Chemical Compositions

The concentrations of DM, CP, EE, CA, NDF, ADF, and HEMI from corn forage just before ensiling were 287, 89.8, 34.9, 50.0, 401, 220, and 186 g/kg, respectively (Table 1). In addition, IVDMD and IVNDFD of corn forage were 661 and 334 g/kg, respectively.

After ensiling, inoculant application did not affect the chemical compositions of corn silage (Table 2). The mean concentrations of DM, CP, EE, CA, NDF, ADF, and HEMI after ensiling were 266, 88.3, 32.8, 51.2, 413, 227, and 185 g/kg, respectively. However, the present study reported that 5M silage and MIX silage had higher IVDMD (*p* = 0.004; 695 and 693 vs. 659 g/kg) and IVNDFD (*p* = 0.006; 403 and 393 vs. 347 g/kg) than those of CON silage, while 6M silage had no differences of IVDMD and IVNDFD among all silages.

### 3.2. Fermentation Characteristics and Microbial Counts

The present study reported that all silages with inoculant applications had lower pH than CON silage after ensiled for 252 d (*p* = 0.008; 3.68, 3.68, and 3.67 vs. 3.73; Table 3). The concentration of lactate was higher in 6M silage and MIX silage than in CON silage (*p* = 0.001; 100.1 and 97.5 vs. 78.3 g/kg), while 5M silage was not different from CON silage. The highest acetate concentration was produced by MIX silage, followed by 5M silage and 6M silage, and then by CON silage (*p* < 0.001; 119.8 vs. 77.3 and 87.3 vs. 49.7 g/kg). Inoculant application did not affect ammonia-N concentration of corn silage. In the present study, propionate and butyrate concentrations were not detected in all silages. The highest ratio of lactate to acetate was produced by CON silage, followed by 5M silage and 6M silage, and then by MIX silage (*p* < 0.001; 1.58 vs. 1.12 and 1.15 vs. 0.81). On the other side, all silages with applied inoculants had higher LAB count (*p* < 0.001; 8.78, 8.83, and 8.77 vs. 7.81 log10 cfu/g) with lower yeast count (*p* = 0.012; 5.57, 5.15, and 5.18 vs. 5.57 log10 cfu/g) and mold count (*p* < 0.001; 3.32, 3.23, and 3.21 vs. 3.68 log10 cfu/g) than those of CON silage.

### 3.3. Rumen Degradation Kinetics

After 72 h of rumen incubation, MIX silage had lower immediately degradable fraction than other silages (*p* < 0.001; 0.69 vs. 0.79, 0.77, and 0.78 mL/g; Table 4). The potentially degradable fraction was higher in MIX silage than in CON silage and 5M silage (*p* = 0.007; 4.33 vs. 3.77 and 3.92 mL/g), while 6M silage had no difference compared to others. Meanwhile, MIX silage had higher total degradable fraction than CON silage (*p* = 0.032; 5.02 vs. 4.56 mL/g), while 5M silage and 6M silage had no difference compared to others. The MIX silage had higher lag phase than 5M silage and 6M silage (*p* = 0.016; 4.11 vs. 3.39 and 3.17 h), while CON silage had no difference compared to other silages. In addition, the fractional degradation rate was similar in all silages.

### 3.4. Rumen Fermentation Indices

In rumen fermentation indices, CON silage had higher pH compared to the other silages (*p* = 0.004; 6.32 vs. 6.28, 6.26, 6.25; Table 5). However, MIX silage had higher total VFA concentration than other silages (*p* = 0.001; 161.0 vs. 138.6, 143.0, and 147.2 mM/L). Propionate concentration was higher in CON silage than 6M silage and MIX silage (*p* = 0.004; 22.0 vs. 20.2 and 20.1%), while 5M silage was not different compared to the other silages. Butyrate concentration was higher in MIX silage than in CON silage and 5M silage (*p* = 0.004; 15.7 vs. 14.6 and 14.8%), while 6M silage had similar concentration to other silages. The 5M silage had higher concentration of iso-valerate than CON silage (*p* = 0.022; 3.20 vs. 2.74%), while 6M silage and MIX silage presented a similar concentration to other silages. Both 6M silage and MIX silage had higher acetate to propionate ratio than CON silage (*p* = 0.009; 2.90 and 2.91 vs. 2.63), while 5M silage had no difference compared to other silages. On the other side, inoculant application did not affect concentrations of ammonia-N and other VFA profiles.

### 3.5. Microbial Changes at Feed-Out Phase

Both without and with contaminations, all silages with applied inoculants extended the growth of LAB by presenting higher (*p* < 0.05) count than CON silage from 24 to 96 h of aerobic exposure (Table 6). The 6M1 silage and MIX silage extended the growth of LAB by presenting higher (*p* < 0.05) LAB count than the other silages at 120 h of aerobic exposure, both without and with contaminations. All silages with applied inoculants both without and with contaminations presented lower (*p* < 0.05) yeast count than CON silage from 24 to 96 h of aerobic exposure. The MIX silage without and with contaminations presented the lowest (*p* < 0.05) yeast count at 120 h of aerobic exposure. Supporting the result of yeast count, all silages with applied inoculants also had lower (*p* < 0.05) mold count than CON silage from 24 to 72 h in treatment without contamination, and from 24 to 48 h in treatment with contamination. Application of 6M silage and MIX silage had lower (*p* < 0.05) mold count than application of CON silage and 5M silage at 96 h in treatment without contamination and at 72 h in treatment with contamination. At 120 h of aerobic exposure, application of MIX silage without contamination presented the lowest (*p* < 0.05) mold count. In general, silages without contamination presented higher LAB count (*p* < 0.001) and lower counts of yeast (*p* = 0.007) and mold (*p* < 0.001) than silages with contamination, and the interaction effect (*p* < 0.001) between inoculant, mold, and aerobic hour were detected on all microbes. In addition, counts of yeast and mold increased linearly (*p* < 0.001) by increasing aerobic hour, while the LAB count decreased linearly (*p* < 0.001).

**Figure 1:**
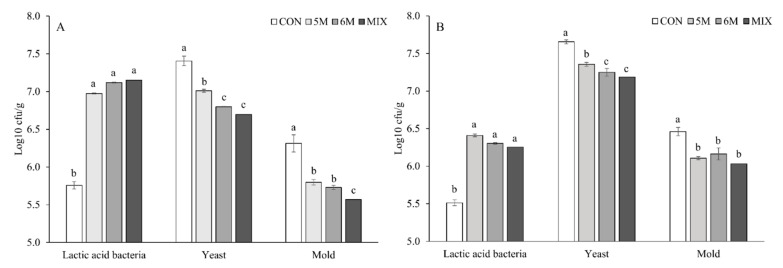
Effects of dual-purpose inoculants on mean count of all microbes from corn silages without (**A**) or with contamination of *Fusarium graminearum* (**B**) during 120 h of aerobic exposure. The mean count was obtained by generating microbial count data from 0 to 120 h. CON, corn silage applied without inoculant; 5M, corn silage applied with *Lactobacillus brevis* 5M2; 6M, corn silage applied with *Lactobacillus buchneri* 6M1; MIX, corn silage applied with the mixture of *L. brevis* 5M2 and *L. buchneri* 6M1 at 1:1 ratio. ^a–c^: Means in same microbe with different superscripts differ significantly (*p* < 0.05). Error bar represents standard error.

The mean count of all microbes was generated from 0 to 120 h of aerobic exposure. In silages without contamination, all silages with inoculant applications presented higher mean count of LAB during feed-out phase than CON silage (*p* < 0.05; 7.02, 7.15, and 7.24 vs. 5.78 log10 cfu/g; Figure 1). Meanwhile, the lowest to the highest mean counts of yeast (*p* < 0.05; 6.70 vs. 6.80 vs. 7.02 vs. 7.40 log10 cfu/g) and mold (*p* < 0.05; 5.56 vs. 5.72 vs. 5.85 vs. 6.31 log10 cfu/g) were produced by MIX silage, 6M silage, 5M silage, and CON silage, consecutively. In silages with contamination, all silages with applied inoculants also had higher mean count of LAB than CON silage (*p* < 0.05; 6.23, 6.37, and 6.32 vs. 5.50 log10 cfu/g). Furthermore, mean counts of yeast and mold in silages with contamination had similar results in silage without contamination. Consecutively, MIX silage, 6M silage, 5M silage, and CON silage had the lowest to highest mean counts of yeast (*p* < 0.05; 7.16 vs. 7.23 vs. 7.39 vs. 7.65 log10 cfu/g) and mold (*p* < 0.05; 6.07 vs. 6.10 vs. 6.17 vs. 6.48 log10 cfu/g).

### 3.6. Aerobic Stability

**Figure 2:**
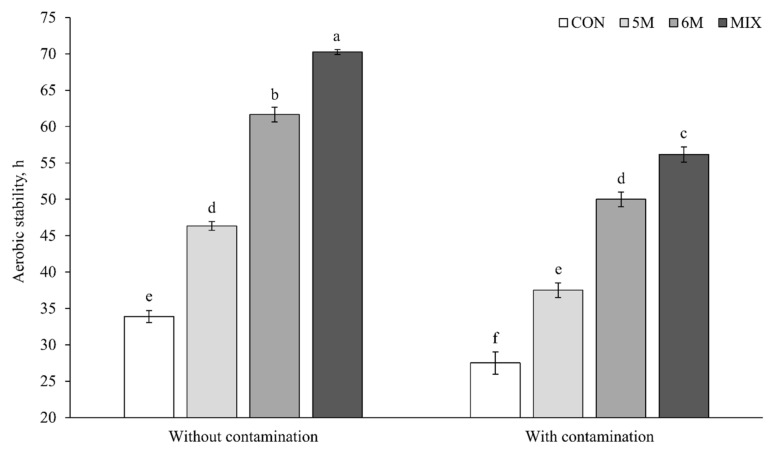
Effects of dual-purpose inoculants on aerobic stability of corn silage. Without contamination, corn silage without *Fusarium graminearum* contamination at silo open; with contamination, corn silage with *F. graminearum* contamination at silo open; CON, corn silage applied without inoculant; 5M, corn silage applied with *Lactobacillus brevis* 5M2; 6M, corn silage applied with *Lactobacillus buchneri* 6M1; MIX, corn silage applied with the mixture of *L. brevis* 5M2 and *L. buchneri* 6M1 at 1:1 ratio. The significant levels of inoculant, *Fusarium*, and inoculant × *Fusarium* are *p* < 0.001, *p* < 0.001, and *p* = 0.05, respectively. ^a–f^ Means with different superscripts differ significantly (*p* < 0.05). Error bar represent standard error.

Generally, silages with contamination had lower aerobic stability than silages without contamination (*p* < 0.001; 42.8 vs. 53.1 h; Figure 2). The highest aerobic stability was produced by MIX silage, followed consecutively by 6M silage, 5M silage, and CON silage both without contamination (*p* < 0.001; 70.3 vs. 61.7 vs. 46.3 vs. 33.9 h) and with contamination (*p* < 0.001; 56.2 vs. 50.0 vs. 37.5 vs. 27.5 h). In addition, the interaction effect between inoculant application and *Fusarium* contamination was reported (*p* = 0.005) in the aerobic stability result. Its interaction presented that 6M silage with contamination had a similar result to 5M silage without contamination (50.0 and 46.3 h) and 5M silage with contamination had a similar result to CON silage without contamination (37.5 and 33.9 h).

## 4. Discussion

### 4.1. Chemical Compositions and Fermentation Characteristics

In the present study, the chemical compositions of *Kwangpyeongok* corn forage were in a normal range according to Lee et al. [24]. After ensiling, chemical compositions of corn silage were not influenced by inoculant application due to the good acidification in all silages. It could be seen from pH and ammonia-N concentration in all silages (Table 3) that they were acidic enough to inhibit nutrient loss and also in a normal range according to Kung et al. [2]. Additionally, the concentrations of CP and EE between before and after ensiling were similar (*p* = 0.346 and *p* = 0.299, respectively; data were not shown in tables), which could indicate less nutrient loss in all silages due to the good ensiling process. Results of IVDMD and IVNDFD during 48 h of rumen incubation in the present study supported our previous study, where application of a mixed *L. brevis* 5M2 and *L. buchneri* 6M1 on farm-scale corn silage could increase nutrient digestibility [16]. Furthermore, corn silage applied single inoculant also presented higher IVDMD and IVNDFD than CON silage that represented activity of carboxylesterase, even though in 6M silage only increased numerically. According to Adesogan et al. [5] and Ribeiro et al. [29], carboxylesterase could break down the lignocellulose, and then increase the accessibility of the other fibrolytic enzyme in the rumen to degrade cellulose or hemicellulose. Generally, group of carboxylesterases can consist of ferulic acid esterase, acetyl xylan esterase, and p-coumaric esterase [29]. The improvement of IVDMD and IVNDFD by inoculant application in the present study was in agreement with our hypothesis that could help to improve silage quality in the field.

In the fermentation characteristics, all silages with inoculant applications presented lower pH than CON silage because they had higher organic acid concentration, such as lactate and acetate [16,30]. An increase of organic acid production in all silages with applied inoculant might be caused by degradation of lignocellulose during ensiling that might provide more soluble carbohydrate. Another reason, an increase of LAB count (Table 3) in all silages with applied inoculants might increase the production of organic acid, and then decrease silage pH lower than CON silage [3,30]. The ammonia-N concentration did not differ among treatments that supported the result of CP concentration after ensiling (Table 2). Ammonia-N concentration in the silage reflects the rate of protein degradation during ensiling [2,30]. With rapid acidification in all silages, undesirable microbes had less chance to degrade the protein content in the silage [2,30]. As the heterofermentative LAB, application of *L. brevis* 5M2 and *L. buchneri* 6M1 could present a lower lactate to acetate ratio on silage, such as in 5M silage, 6M silage, and MIX silage. This could have occurred because heterofermentative LAB have the ability to convert lactate into acetate [3,30]. Otherwise, CON silage presented higher lactate to acetate ratio indicating that the fermentation process might be controlled by homofermentative LAB as epiphytic bacteria. In addition, acetate has antifungal activity to inhibit undesirable microbes in the silage [3,31]. The investigation of our previous study found that molds still could grow in the corn silage although they had high lactate concentration and low pH [31]. It could occur because of the low production of antifungal substance, such as acetate during ensiling [31]. Thus, the presence of antifungal inoculant was necessary for corn silage production. Interestingly, application of a mixed inoculant presented a higher acetate concentration than a single inoculant. According to Santos et al. [32], the use of mixed LAB, such as in the present study, would cause a multi-microbial process during ensiling that combines beneficial effects of each LAB species. Due to its higher beneficial effects, nowadays, many manufacturers produce commercial inoculant containing mixed LAB [32]. On the other opinion, higher acetate concentration in the MIX silage might be caused by the beneficial relation among *L. brevis* 5M2 and *L. buchneri* 6M1 such as reported by previous study [33]. Nevertheless, Vinderola et al. [33] explained that application of a mixed LAB for inoculant did not always result in beneficial effects for ensiled product, where its effect was dependent on genus or species of LAB. In microbial count, decreased counts of yeast and mold in 5M silage, 6M silage, and MIX silage were caused by higher acetate concentration in these silages than in CON silage, which supported the result of previous studies [3,16,31]. In the present study, application of all inoculant treatments on corn silage increased LAB count that showed a similar result with Paradhipta et al. [16]. However, other studies reported that application of microbial additives did not always promise to increase LAB count on silage [18,34].

### 4.2. Rumen Degradation

Application of a mixture of *L. brevis* 5M2 and *L. buchneri* 6M1 on corn silage decreased the immediately degradable fraction that is associated with the increase of organic acid concentration such as lactate and acetate. The immediately degradable fraction consists of soluble carbohydrates [28,35] that are used by LAB to produce organic acids during ensiling [30]. Thus, high utilization of soluble carbohydrates to synthesize organic acids during ensiling would decrease concentration of immediately degradable fraction. Supporting the results of IVDMD and IVNDFD, carboxylesterase activity from *L. brevis* 5M2 and *L. buchneri* 6M1 improved the total degradable fraction in all silages with applied inoculants, even though in 5M silage and 6M silage it only numerically increased. These results were also in agreement with several previous studies that reported an improvement on nutrient digestibility of silage by applying LAB producing ferulic acid esterase [8,36]. The application of these new inoculants could be applied to solve the problem with low digestibility of silage in the field. However, the other studies reported that application of LAB producing ferulic acid esterase on fermentation of forage did not always increase nutrient digestibility of silages [18,19]. In the present study, the carboxylesterase was effective only in MIX silage to increase the potentially degradable fraction and the total degradable fraction. Similar to the result of acetate, beneficial relation between *L. brevis* 5M2 and *L. buchneri* 6M1 might be a reason for these results of degradable fraction.

The improvements of the potentially degradable fraction and the total degradable fraction on MIX silage were also supported with the result of total VFA concentration in the present study, where MIX silage was also the only one that improved total VFA concentration. An increase of the total degradable fraction is associated with an increase of total VFA concentration in the rumen [35]. Furthermore, a decrease of rumen pH in MIX silage resulted in an increase of VFA concentration that was in agreement with Chesson and Forsberg [35]. Nevertheless, application of a single inoculant on the corn silage also numerically increased total VFA concentration that could contribute to decreasing rumen pH lower in 5M silage and 6M silage than in CON silage. The higher butyrate concentration in MIX silage was associated with lower propionate concentration, which supported the result of previous studies [35,37]. In addition, the high butyrate concentration in the rumen reflected high degradation of structural carbohydrate. This indicated that application of mixed *L. brevis* 5M2 and *L. bucnheri* 6M1 increased the degradation of structural carbohydrate of corn silage that confirmed activity of carboxylesterase.

### 4.3. Aerobic Deterioration

In the present study, contaminating *F. graminearum* on corn silage at silo open stimulated the growth of mold and yeast that accelerated the decay of silage and decrease of LAB count during feed-out phase. The LAB count was decreased linearly by a longer time of aerobic exposure because the presence of oxygen could inhibit its growth as anaerobic bacteria [3,30]. However, inoculant application could extent the growth of LAB count on corn silage both without and with contaminations. This result supported our previous study that inoculation of baled corn silage with a mixed *L. brevis* 5M2 and *L. buchneri* 6M1 also extended the growth of LAB during feed-out phase [16]. The *L. brevis* 5M2 and *L. buchneri* 6M1 might have higher aerobic tolerance than epiphytic LAB grown in corn silage.

As aerobic microorganisms, yeast and mold counts were increased linearly by longer time of aerobic exposure due to the presence of oxygen [10,30]. In the present study, longer time of aerobic exposure and *Fusarium* contamination decreased shelf life of corn silage after silo opening. Both without and with *Fusarium* contamination, application of all inoculants on corn silage could inhibit the aerobic deterioration at feed-out phase due to the presence of antifungal activity. Previous research also reported a similar result to the present study that application of LAB producing antifungal substances on silages at ensiling inhibited the growth of yeasts and molds after silo opening [7,34]. Generally, the effectiveness of inoculant to inhibit undesirable microbes in the silage could decrease by *Fusarium* contamination and longer time of aerobic exposure. However, as the result of an interaction effect among independent variables (inoculant application, *Fusarium* contamination, and aerobic hour), MIX silage with contamination inhibited yeasts and molds better than CON silage without contamination until 96 h of aerobic exposure. Moreover, it presented similar inhibition of yeasts and molds to 6M silage without contamination. Supporting the result of aerobic stability, application of a mixed inoculant presented greater inhibitions of yeasts and molds than a single inoculant at feed-out phase. It also could be seen that MIX silage presented the lowest mean counts of yeast and mold during 120 h of aerobic exposure (Figure 2). On the other side, application of *F. graminearum* on corn silage was reported to increase the yeast growth at feed-out phase. The reason for this result was unclear. As generally known, yeast and mold are classified in the same group as fungi [10,30]. A symbiotic relation between *F. graminearum* and epiphytic yeast in the corn silage might influence the result of increasing yeast count at feed-out phase in silage with contamination.

The change of microbial counts at feed-out phase influences the aerobic stability of silage [2]. Contamination of *F. graminearum* decreased the aerobic stability of corn silage in the present study that associated with the increase growths of yeast and mold (Table 4). The results of present study indicated that all inoculants both as single and mixed confirmed antifungal activity by increasing aerobic stability of corn silage both without and with contaminations. Supported by the result of acetate, MIX silage presented the highest aerobic stability, followed by 6M silage and 5M silage. Moreover, MIX silage and 6M silage with *F. graminearum* contamination still had higher aerobic stability than CON silage without contamination. As the explained before, acetate is one of the antifungal compounds that was reported to increase aerobic stability of silage [3,31]. The result of aerobic stability in the present study supported Kleinschmit et al. [6] and Liu et al. [7] that also reported a similar effect using LAB producing antifungal activity. The result of antifungal activity by LAB in the present study was in agreement with the result of antifungal activity by essential oil [38,39]. The application of essential oil on the feed or food could control the growth of pathogenic microorganisms during aerobic condition [38]. Similar with antifungal activity by LAB, the essential oil also generally contains antibacterial and antifungal substances that can inhibit several aerobic microbes including Gram-positive or -negative bacteria and yeast [39].

Almost all of the corn silages ended the aerobic stability when the yeast count was higher than 6.00 log10 cfu/g both without and with contamination. Furthermore, the mold grew massively after aerobic stability was ended in all silages. It could indicate that the increase of silage temperature was initiated by yeast in the present study. Similar to the present study, Kung et al. [2] also explained that aerobic stability of corn silage could be zero if yeast count was more 6.00 log10 cfu/g juts after silo opening. According to Lindgren et al. [40], yeast initiates deterioration and the increase of silage temperature after silo opening. Generally, shelf life of silage in the field decreases along with longer feed-out phase due to the increase of yeast and mold growths. The extended shelf life of corn silage by inoculant application supported our hypothesis that antifungal producing LAB presented a beneficial effect on shelf life of corn silage. In addition, application of these new inoculants could help to improve silage management in the field, especially against the contamination of mold.

## 5. Conclusions

In the present study, application of *L. brevis* 5M2 and *L. buchneri* 6M1 as single and mixed inoculant showed beneficial effects on corn silage by improving fermentation characteristics and inhibiting growth of undesirable microbes. In rumen degradation, application of *L. brevis* 5M2 and *L. buchneri* 6M1 on corn silage as a mixed inoculant presented better carboxylesterase effects than as a single inoculant to improve degradable fractions and total VFA concentration. Contamination of *F. graminearum* at feed-out phase increased aerobic deterioration of corn silage, but application of all inoculant treatments presented antifungal effects by extending shelf life of corn silage during feed-out phase. Therefore, the present study concluded that application of mixed *L. brevis* 5M2 and *L. buchneri* 6M1 on corn silage production is recommended for both activities of antifungal and carboxylesterase by extending shelf life at feed-out phase and improving rumen degradation, respectively.

## Figures and Tables

**Table 1:** The chemical composition of fresh forage before ensiling (g/kg, DM).

Item ^1^	Forage	SE
Dry matter	287	0.431
Crude protein	89.8	0.100
Ether extract	35.1	0.208
Crude ash	49.8	0.192
Neutral detergent fiber	404	1.070
Acid detergent fiber	219	0.902
Hemicellulose	186	1.884
IVDMD	661	2.058
IVNDFD	334	2.251

^1^: IVDMD, in vitro dry matter digestibility; IVNDFD, in vitro neutral detergent fiber digestibility.

**Table 2:** Effects of dual-purpose inoculants on chemical compositions of corn silage ensiled for 252 d (g/kg, DM).

Item ^1^	Treatment ^2^				SEM	*p*-Value
	CON	5M	6M	MIX		
Dry matter	263	268	267	266	2.115	0.088
Crude protein	87.9	88.5	88.5	88.2	1.451	0.948
Ether extract	30.3	33.2	35.6	32.2	3.839	0.421
Crude ash	51.8	49.8	50.5	52.7	4.410	0.812
Neutral detergent fiber	415	416	406	418	13.57	0.881
Acid detergent fiber	230	227	228	223	6.443	0.673
Hemicellulose	185	189	180	185	13.32	0.840
IVDMD	659 ^b^	695 ^a^	676 ^a b^	693 ^a^	6.620	0.004
IVNDFD	347 ^b^	403 ^a^	375 ^a b^	393 ^a^	14.05	0.006

^a,b^: Means in the same row with different superscripts differ significantly (*p* < 0.05). ^1^: IVDMD, in vitro dry matter digestibility; IVNDFD, in vitro neutral detergent fiber digestibility. ^2^: CON, corn silage applied without inoculant; 5M, corn silage applied with *Lactobacillus brevis* 5M2; 6M, corn silage applied with *Lactobacillus buchneri* 6M1; MIX, corn silage applied with the mixture of *L. brevis* 5M2 and *L. buchneri* 6M1 at 1:1 ratio.

**Table 3:** Effects of dual-purpose inoculants on fermentation characteristics and microbial count of corn silage ensiled for 252 d.

Item	Treatment ^1^				SEM	*p*-Value
	CON	5M	6M	MIX		
Fermentation characteristics						
pH	3.73 ^a^	3.68 ^b^	3.68 ^b^	3.67 ^b^	0.018	0.008
Ammonia-N, g/kg DM	0.88	0.81	0.78	0.83	0.040	0.061
Lactate, g/kg DM	78.3 ^c^	86.2 ^b c^	100.1 ^a^	97.5 ^a b^	4.798	0.001
Acetate, g/kg DM	49.7 ^c^	77.3 ^b^	87.3 ^b^	119.8 ^a^	12.15	<0.001
Propionate, g/kg DM	ND ^2^	ND	ND	ND	N/A	N/A
Butyrate, g/kg DM	ND	ND	ND	ND	N/A	N/A
Lactate: acetate	1.58 ^a^	1.12 ^b^	1.15 ^b^	0.81 ^c^	0.065	<0.001
Microbial count, log10 cfu/g						
Lactic acid bacteria	7.81 ^b^	8.78 ^a^	8.83 ^a^	8.77 ^a^	0.109	<0.001
Yeast	5.57 ^a^	5.15 ^b^	5.18 ^b^	5.13 ^b^	0.164	0.012
Mold	3.68 ^a^	3.32 ^b^	3.23 ^b^	3.21 ^b^	0.097	<0.001

^a–c^: Means in the same row with different superscripts differ significantly (*p* < 0.05). ^1^: CON, corn silage applied without inoculant; 5M, corn silage applied with *Lactobacillus brevis* 5M2; 6M, corn silage applied with *Lactobacillus buchneri* 6M1; MIX, corn silage applied with the mixture of *L. brevis* 5M2 and *L. buchneri* 6M1 at 1:1 ratio. ^2^: ND, not detected.

**Table 4:** Effects of dual-purpose inoculants on in vitro digestibility and degradation kinetics of corn silage incubated with rumen buffer for 72 h.

Item ^1^	Treatment ^2^				SEM	*p*-Value
	CON	5M	6M	MIX		
A, mL/g DM	0.79 ^a^	0.77 ^a^	0.78 ^a^	0.69 ^b^	0.012	<0.001
B, mL/g DM	3.77 ^b^	3.92 ^b^	4.05 ^a b^	4.33 ^a^	0.099	0.007
A + B, mL/g DM	4.56 ^b^	4.69 ^a b^	4.83 ^a b^	5.02 ^a^	0.211	0.032
C, %/h	0.14	0.13	0.12	0.12	0.007	0.206
L, h	3.58 ^a b^	3.39 ^b^	3.17 ^b^	4.11 ^a^	0.230	0.016

^a,b^: Means in the same row with different superscripts differ significantly (*p* < 0.05). ^1^: A, the immediately degradable fraction; B, the potentially degradable fraction; A+B, the total degradable fraction; C, the fractional degradation rate; L, the lag phase. ^2^: CON, corn silage applied without inoculant; 5M, corn silage applied with *Lactobacillus brevis* 5M2; 6M, corn silage applied with *Lactobacillus buchneri* 6M1; MIX, corn silage applied with the mixture of *L. brevis* 5M2 and *L. buchneri* 6M1 at 1:1 ratio.

**Table 5:** Effects of dual-purpose inoculants on rumen fermentation indices of corn silage incubated with rumen buffer for 72 h.

Item ^1^	Treatment ^2^				SEM	*p*-Value
	CON	5M	6M	MIX		
pH	6.32 ^a^	6.28 ^b^	6.26 ^b^	6.25 ^b^	0.014	0.004
Ammonia-N, mg/dL	35.0	35.2	34.3	33.4	1.310	0.318
Total VFA, m*M*/L	138.6 ^b^	143.0 ^b^	147.2 ^b^	161.0 ^a^	4.668	0.001
Acetate, % molar	57.8	57.7	58.6	58.4	0.552	0.089
Propionate, % molar	22.0 ^a^	21.3 ^a,b^	20.2 ^b^	20.1 ^b^	0.654	0.004
Iso-butyrate, % molar	1.25	1.35	1.34	1.29	0.130	0.688
Butyrate, % molar	14.6 ^b^	14.8 ^b^	15.3 ^a,b^	15.7 ^a^	0.337	0.004
Iso-valerate, % molar	2.74 ^b^	3.20 ^a^	3.08 ^a,b^	3.03 ^a^	0.181	0.022
Valerate, % molar	1.56	1.63	1.49	1.44	0.221	0.629
Acetate: propionate	2.63 ^b^	2.70 ^a,b^	2.90 ^a^	2.91 ^a^	0.116	0.009

^a,b^: Means in the same row with different superscripts differ significantly (*p* < 0.05). ^1^: VFA, volatile fatty acid. ^2^: CON, corn silage applied without inoculant; 5M, corn silage applied with *Lactobacillus brevis* 5M2; 6M, corn silage applied with *Lactobacillus buchneri* 6M1; MIX, corn silage applied with the mixture of *L. brevis* 5M2 and *L. buchneri* 6M1 at 1:1 ratio.

**Table 6:** Effects of dual-purpose inoculants on microbial counts of corn silage without or with *Fusarium* contamination at feed-out phase (log 10 cfu/g).

Item	Without Contamination ^1^				With Contamination				SEM
	CON	5M	6M	MIX	CON	5M	6M	MIX	
Lactic acid bacteria									
24 h	7.19 ^c^	8.04 ^b^	8.51 ^a^	8.21 ^b^	7.11 ^c^	7.83 ^b^	7.93 ^b^	7.97 ^b^	0.103	
48 h	6.23 ^c^	7.81 ^a^	7.38 ^b^	8.02 ^a^	5.23 ^d^	6.12 ^c^	6.28 ^c^	6.28 ^c^	0.137	
72 h	5.42 ^b^	8.17 ^a^	8.50 ^a^	8.35 ^a^	4.74 ^c^	5.41 ^b^	5.52 ^b^	5.28 ^b^	0.168	
96 h	4.16 ^c^	5.11 ^b^	5.80 ^a^	5.86 ^a^	4.06 ^c^	5.05 ^b^	5.52 ^a b^	5.28 ^b^	0.137	
120 h	3.88 ^c^	4.19 ^b c^	4.29 ^b^	4.68 ^a^	4.04 ^b c^	4.21 ^b c^	4.13 ^b c^	4.36 ^a b^	0.143	
Yeast									
24 h	5.55 ^c^	5.19 ^d^	5.19 ^d^	5.10 ^d^	6.25 ^a^	5.81 ^b^	5.75 ^b c^	5.58 ^b c^	0.094	
48 h	6.91 ^b^	6.35 ^d^	5.94 ^e^	5.89 ^e^	7.43 ^a^	7.37 ^a^	6.77 ^b c^	6.67 ^c^	0.073	
72 h	7.98 ^b c^	7.52 ^d^	6.93 ^e^	6.96 ^e^	8.29 ^a^	8.00 ^b^	7.80 ^c^	7.85 ^b c^	0.083	
96 h	9.12 ^a^	8.81 ^b c^	8.46 ^c d^	8.36 ^d^	9.17 ^a^	8.90 ^b^	8.70 ^b c^	8.61 ^c^	0.087	
120 h	9.27 ^a^	9.09 ^a^	9.13 ^a^	8.76 ^b^	9.17 ^a^	9.13 ^a^	9.19 ^a^	9.14 ^a^	0.058	
Mold									
24 h	3.74 ^a^	3.27 ^c^	3.30 ^b c^	3.25 ^c^	3.83 ^a^	3.39 ^b c^	3.49 ^b^	3.51 ^b^	0.093	
48 h	4.92 ^b^	4.33 ^c^	4.38 ^c^	4.43 ^c^	5.52 ^a^	5.02 ^b^	4.92 ^b^	5.03 ^b^	0.103	
72 h	6.92 ^b^	6.04 ^c^	5.64 ^d^	5.67 ^d^	7.29 ^a^	7.04 ^a b^	6.75 ^b^	6.76 ^b^	0.124	
96 h	9.14 ^a^	8.97 ^a b^	8.50 ^c^	7.94 ^d^	9.08 ^a b^	9.04 ^a b^	8.92 ^b^	8.52 ^c^	0.089	
120 h	9.43 ^a^	9.15 ^a^	9.23 ^a^	8.85 ^b^	9.43 ^a^	9.23 ^a^	9.30 ^a^	9.36 ^a^	0.120	
Contrast ^2^			Lactic acid bacteria			Yeast		Mold	
INO			<0.001			<0.001		<0.001	
FUS			<0.001			0.007		<0.001	
HOUR			<0.001			<0.001		0.001	
INO*FUS			<0.001			<0.001		0.001	
INO*HOUR			<0.001			<0.001		0.001	
FUS*HOUR			<0.001			<0.001		<0.001	
INO*FUS*HOUR			<0.001			<0.001		<0.001	
HOUR linear			<0.001			<0.001		<0.001	
HOUR quadratic			0.679			0.617		0.412	

^a–e^: Means in the same row with different superscripts differ significantly (*p* < 0.05). ^1^: Without contamination, corn silage without *Fusarium graminearum* contamination at silo open; with contamination, corn silage with *F. graminearum* contamination at silo open; CON, corn silage applied without inoculant; 5M, corn silage applied with *Lactobacillus brevis* 5M2; 6M, corn silage applied with *Lactobacillus buchneri* 6M1; MIX, corn silage applied with the mixture of *L. brevis* 5M2 and *L. buchneri* 6M1 at 1:1 ratio. ^2^: INO, inoculant effect; FUS, *Fusarium* effect; HOUR, aerobic hour effect; INO*FUS, interaction effect between inoculant and *Fusarium*; INO*HOUR, interaction effect between inoculant and aerobic hour; FUS*HOUR, interaction effect between *Fusarium* and aerobic hour; INO*FUS*HOUR, interaction effect between inoculant, *Fusarium*, and aerobic hour.

## Data Availability

Data are available on request from the corresponding author with reasonable reason.
